# Towards a Feminist Geo-legal Ethic of Caring Within Medical Supply Chains: Lessons from Careless Supply During the COVID-19 Pandemic

**DOI:** 10.1007/s10691-023-09520-1

**Published:** 2023-02-21

**Authors:** Ania Zbyszewska, Sharifah Sekalala

**Affiliations:** 1 Department of Law and Legal Studies, Carleton University, Ottawa, Canada; 2 School of Law, Warwick University, Coventry, UK

**Keywords:** COVID-19 pandemic, Ethics of care, Feminist geo-legality, Medical supply chains, Pandemic preparedness

## Abstract

The COVID-19 crisis illustrates the fragility of supply chains. Countries with excellent health systems struggled to ensure essential supplies of food, medicines, and personal protective equipment which were vital to a fast and effective response. Using geo-legality, which maps the constitutive relations between law and space, we argue that the failure of supply chains in many western countries during the crisis reveals a fundamental tension between their role as facilitators of care and caring, and the logistic logics by which they operate. While supply chains link the intimate, domestic concerns of providing medical care with the globalised geographical concerns of moving goods across different jurisdictions at the right time, their contemporary organisation and regulation does not reflect the caring relations and public goods they are meant to support. Drawing on analysis of examples from Canada, the United Kingdom, and the United States, this article argues that a reconfiguration of supply chains in accordance with feminist approaches that place care at the centre of supply chain operation and organisation will be important to amendments of both domestic and global health law.

## Introduction

The COVID-19 pandemic exposed the fragility of global supply chains. Shortages of essential supplies of food, medicines, and personal protective equipment (PPE) undermined fast and effective responses even in countries with otherwise robust health care systems. Considering the extent of this crisis, significant attention has been directed towards exploring alternatives to how medical supply chains are governed, improving their efficiency through technological means, or developing new ones at the national scale (Gereffi 2020; O’Leary 2020; O’Neil 2020). What has been questioned less often, however, are the logics according to which these chains operate. Drawing on feminist scholarship in legal and critical geography, this paper explores the geo-legal constitution of modern supply chains (Bricknell and Cuomo 2017), focusing on the fundamental tension between the logistical logics that animate them and the role these chains play in facilitating caring relations and delivery of public goods such as health care. We argue that recognising and addressing this tension is essential to developing new and more care-facilitating ways of governing and organising medical supply chains.

Given their dispersed organisational structure and the fact that they traverse jurisdictions, supply chains exemplify what critical scholars working in law and geography refer to as geo-legality. Geo-legality is a concept intended to capture the co-generative relations between law and space on the one hand, and geopolitics and geoeconomics on the other (Smith et al. 2014). In feminist scholarship, geo-legality is further nuanced to encompass the intimate and the everyday concerns in the category of geopolitics (Bricknell and Cuomo 2017), thereby connecting the spaces and politics operating at multiple scales, ranging from the intimate to the global. As such, geo-legality, especially its feminist understanding, can be used to conceptualise medical supply chains’ organisation and governance in a way that centres their relational character, since these chains play a vital role in linking the domestic concerns of providing health care services with the globalised concerns of distributing medical goods across different jurisdictions. However, critical scholarship on supply chains suggests that supply chains are governed by and perpetuate logistical, militaristic, and postcolonial extractive logics (Cowen 2014; Alessandrini 2020) which tend to be incompatible with the practices and relations of health care. What happens when these logics also inform systems of pandemic preparedness and when they drive pandemic responses?

This article traces the geo-legality of government-constructed medical supply chains and their implications for care delivery before and during the ongoing COVID-19 pandemic. In our analysis, we focus on examples from Canada, the United Kingdom (UK), and the United States (US). Through both the World Health Organization (WHO) Index and the Global Health Security Index (GHS Index), these three countries were deemed, before the pandemic, to be amongst some of the best prepared for a crisis. In all three, however, failure of national strategic stockpiles and of supply chains resulted in severe shortages of medical goods, especially of PPE, in the first phase of the pandemic. These shortages impeded effective care delivery, with secondary care settings such as long-term care and nursing homes being especially impacted. In our analysis, we use these three country examples to examine the operational and governance choices related to supply chains, their underlying logics, and their consequences for care delivery at three stages: pandemic preparedness, pandemic response, and post-pandemic resilience planning. All three countries were advocates of global trade integration and embraced neoliberalism to varying degrees. We use them in an illustrative way to analyse the failure of countries to provide medical health supplies for care workers and then use a feminist ethic of care analysis to reflect on the paucity of proposed solutions and to offer alternative ways in which we could structure supply chains after the current pandemic.

The paper is organised as follows. Following a brief explanation of feminist geo-legality, we discuss the problem that the tensions inherent in the differentiated geography of essential medical supply production and sourcing along supply chains posed at the outset of the pandemic and consider how law is implicated in the reproduction of mainstream approaches to managing health crises or emergencies. Next, we analyse the geo-legality of government-constructed medical supply chains and their implications for care delivery before and during the COVID-19 pandemic in Canada, the UK, and the US, and at different sites of the care continuum (i.e. acute care and hospitals vs care homes and spaces of perceived lower-value care). We conclude with a reflection on the feminist ethic of care—as a lens for evaluation of future-oriented proposals and an alternative and transformational logic for organising and governing the legal geographies of medical supply. Specifically, we explore how the feminist ethic of care can ground alternatives to the current organisation of medical supply in a way that attends also to different forms of labour that are involved in the global production and circulation of medical supply. As we suggest, prioritising care in medical supply chains necessitates reparative justice and better redistribution of resources, because proposals for reorganisation come with costs and resource implications that affect capacities to respond to a crisis in a geopolitically uneven way.

## Feminist Geo-legality of Supply Chains

Developed in legal geography, geo-legality is a concept that links spatio-legal concerns with geopolitics and geoeconomics (Smith 2014). As critical geographers Cowen and Smith (2009) observe, the latter two represent technologies and ideologies central to the historic constitution of global political, economic, and cultural geographies organised by nation states and, increasingly, markets, with the two in tension yet co-generative of each other. Geo-legality broadens this argument to law, geopolitics, and geoeconomics.

While geo-legality analysis tends to focus on law’s global, transnational, or international operations, viewed through a feminist lens, geo-legality also encompasses law’s imbrication with intimacy and the everyday concerns that are sometimes left out of studies of global dynamics (Bricknell and Cuomo 2017). Rachel Pain and Lynn Staeheli (2014, 345) conceptualise intimacy as an intersection of (1) spatial relations stretching from proximate to distant/global, (2) modes of interaction that (may) stretch from personal to distant/global, and (3) practices that apply to but also connect the body (or the home, or other intimate sites) to that which is distant or global. Crucially for them, intimacy is not just affected by developments at the geo/global scale but is in fact integral to the latter’s constitution: the global is also and *already* intimate. Similarly, the feminist approach to geo-legality intermeshes intimacy and everyday concerns with the spatio-legal processes taking place at the global scale into a single yet multiscalar complex (Bricknell and Cuomo 2017).

Here, we use feminist geo-legality to capture the complex political, economic, and governance developments that have positioned supply chains as integral to global and local pandemic preparedness strategies, and thus, to the delivery of care in a crisis. Importantly, as critical scholars have shown, supply chains are not neutral conduits but rather operate on specific logics. Along with ‘just-in-time’ delivery systems, supply chains are amongst geoeconomic forms that first emerged to address problems of national security and geopolitics (Cowen and Smith 2009). Rooted in the field of US military procurement and logistics, supply chains are seen as a mechanism for inserting logics of capitalism and security into all sites (Cowen 2014), in ways that maintain, or extend, postcolonial orthodoxy and extraction of value (Alessandrini 2020).^1^ By linking states, capital, and labour through the chain form, supply chains supercharge the extractive operations of capital by allowing it to take advantage of, and in turn command from the ‘outside’, heterogeneous productive environments, including the material conditions of labour and social reproduction (Mezzadra and Neilson 2017, 198). At the same time, supply chains have been positioned^2^ as a mechanism for circulating goods and commodities to sustain populations^3^ or, as we might add, to respond to global health emergencies. Provision of health care, for instance, is not possible without the supply of care-facilitating goods and commodities at the high end (i.e. medicines and medical equipment, including ventilators) and low end (i.e. PPE, including gloves, gowns, and masks) of the spectrum (Gereffi 2020). This makes supply chains the vital link between sites where these goods are made and the sites where they are needed to deliver or provide care. How consequential to this key task are the logistical and extractive logics that supply chains are governed by and perpetuate?

In our analysis of the role that supply chains played prior to and during the pandemic, we see the ways in which the logistical logics operated to reproduce the globalised medical supply system despite its failures and did so with particular consequences for relations and practices of care in a crisis. Our preliminary analysis of the proposals to make supply chains more resilient reveals that they too, for the most part, are suffused with these very logics. We turn to this three-step analysis next, beginning with the geo-legal constitution of pandemic preparedness, and the role of supply chains therein.

## The Pre-pandemic World: Neoliberal Geo-legality, Preparedness, and Failure of Medical Supply Chains

Developing robust supply chains is an essential component of preparedness for pandemics. However, as we illustrate in this section, the dominant approach to preparedness is embedded in neoliberal geo-legality, at the core of which are policy and governance frameworks that have positioned trade and just-in-time circulation of medical goods through global supply chains as central to achieving health outcomes. Adherence to this approach left apparently well-prepared countries with massive shortages of supplies essential to deliver care in a crisis, especially in secondary care settings.

## Two Different Regimes, Same Logic

Two international regulatory regimes which govern medical supply chains are key to our analysis. These are, first, the rules aimed at curbing infectious diseases under the International Health Regulations (IHR) and, second, the WTO rules which govern the movement of trade and services.

The purpose of the IHR is to prevent, protect against, control, and provide a public health response to the international spread of disease in ways that are commensurate with public health risks, to minimise interference with international traffic and trade (IHR 2005). The IHR therefore seem to normatively connect health outcomes to trade interests, and this is further reinforced by a range of specific IHR provisions. For example, while the regulations contain a number of provisions on supply chains and stockpiling, these are subordinate in scope to the bulk of the agreement that focuses on international trade and traffic. These provisions are described in the IHR as ‘core capacities’ and are meant to ensure that countries can monitor and respond adequately to public health crises. Issues around the supply chains and stockpiles are included in national emergency health response plans (IHR 2005, Annex 1). However in Annex B, these capacities are tackled through mechanisms that for the most part facilitate trade, with emphasis on travellers, on inspections for ships and flights, and on ports of entry. Additionally, Article 57 of the IHR makes it clear that states need to comply with obligations from other treaties which, of course, includes trade treaties and also specifically allows states to apply common rules for regional, economic integration, which for many regional blocs has led to trade-offs that prioritised trade interests at the expense of preparedness for pandemics.

Increased globalisation has been accompanied by proliferation of multilateral trade treaties, such as those of the WTO. Within the current WTO rules, countries have several ways to ensure that they are maintaining medical supply chains. First, countries can use free trade agreements to ensure that they have preferential trading partners that reduce tariffs on essential products such as ventilators and other medical supplies. Second, governments can stockpile critical supplies so that they are prepared for crises (Meyer 2020). Some states such as Norway have used these sorts of exceptions to stockpile supplies for up to two years in order to prepare for pandemics (Heiskanen et al. 2017). Other exceptions also allow states to impose temporary export bans on essential supplies in order to protect human health during a crisis (Meyer 2020). Therefore, although the multilateral trade regime ostensibly allows states to structure supply chains in ways that may shield *medical* supply chains, the ways in which global trade architecture is arranged reinforces the dominance of trade. Medical supply chains therefore become a small and often neglected part amidst broader trading preferences.

## Neoliberalism and the Logics of Logistics

Thus, we see that law plays a key role in positioning trade as central to medical supply and to forging the required links between different geographical locations through multilateral and other trading systems. In turn, states ultimately promote the privatisation of public goods (Slobodian 2018). The legal application of trade rules reduces states’ autonomy in making decisions on medical supply chains, because in many instances states rely on interlinked value chains in which different components for medical supplies are manufactured in different geographical locations. Additionally, the increased privatisation of medical supplies which should be public goods means that governments are not entirely in control of public procurement and rely on private companies who prefer a just-in-time model to supply goods at the cheapest price possible in order to remain profitable.

From the early 1980s this led to manufacturing production being increasingly structured in what is now known as global supply chains (or global value chains) through processes that minimise costs including of labour and manufacturing, as well as tax impacts. According to neoliberal orthodoxy, an increased number of workers in the world market was to ensure access to cheaper imports as well as economic growth in developing countries (Stiglitz 2003). China, for instance, became a critical part of many global supply chains—both as a manufacturer and assembler and as a consumer—due to its huge workforce and emerging affluent population.

The medical goods global market also exhibits pronounced geographical differentiation that supply chains rely upon and reproduce to extract value. Many developed countries such as the US and Germany have developed a specialised high-tech medical devices sector, while low-cost hubs such as China and Malaysia became leading producers of PPE such as gloves and masks (Gereffi 2020, 289). With preparedness strategies reliant on the assumption of logistical coordination through global supply and just-in-time models, this geographical specialisation posed particular problems for countries as supply chains failed, affecting access to medical goods not readily accessible domestically. This was compounded by the failure of these states to prepare by maintaining strategic stockpiles of goods they sourced through global supply chains, so as to avoid storing ‘excessive’ amounts of inventory (Gereffi 2020).

These supply and preparedness failures stand in contrast to the assumptions and assessments about states’ capacity to deal with health crises that we noted earlier. For one, the view that crises are exceptional and largely located within a narrow geographical space, often in developing countries, has been very influential on how preparedness is approached and assessed. Thus, within the context of the IHR, the WHO has very much focused on supply chains in low- and middle-income countries, with an emphasis on providing emergency assistance for developing countries which do not have robust supply chains to respond to health emergencies (WHO 2015). This led to the erroneous assumption that developed countries had high levels of preparedness in their medical supply chains.

Although the WHO had a tool to assess countries’ preparedness capabilities, more credence was given to the GHS Index, a private tool prepared by the Johns Hopkins Center for Health Security, the Nuclear Threat Initiative (NTI) and the Economist Intelligence Unit (EIU) (GHS Index 2019; Kandel et al. 2020). In 2019 the GHS Index developed a comprehensive assessment of 195 states’ preparedness for potential pandemics, classifying countries from the most prepared to the least prepared. Although, overall, the assessment found that most countries were unprepared, our three case study countries were deemed as some of the most prepared, with the US scoring 83.5 (in first place), the UK 77.9 (in second place), and Canada 73.5 (in fifth place) (GHS Index 2019). Given that these countries were also amongst the worst affected during the first phase of the pandemic, with an extremely high number of cases and deaths, this categorisation has now been questioned (Abbey et al. 2020; Horton 2020; Kandel et al. 2020; Razavi et al. 2020; Timmis and Brüssow 2020).

## Neoliberal Geo-legality and the Failure of Supply Chains in Canada, the UK, and the US

As we discussed above, the pre-pandemic phase was characterised by the assumption of preparedness refracted through neoliberal geo-legality that positioned just-in-time procurement of supplies through global supply chains as key in responding to crises. This lack of *actual* preparedness led to a collapse of the PPE market at the onset of the pandemic, which, as we explore in next section, had a disproportionate impact on those who provided intimate care, especially in the so-called secondary frontline (i.e. those in care homes).^4^

In all three countries, there was a culture of decentralising national stockpiles due to chronic underfunding. This led to a fragmented supply landscape for the provision of medical supplies, especially PPE. The US, UK, and Canada all boasted of some stockpiles of PPE before the crisis began (National Academies of Sciences, Engineering, and Medicine 2016; Laing and Westervelt 2020; Braga 2021). However, these stockpiles had been considerably diminished due to outsourcing of PPE in all three jurisdictions which prioritised cost-effectiveness of storage. For instance, the UK relied on the NHS Supply Chain, which in turn outsourced its stockpiling functions to the just-in-time model provided by numerous private companies such as the parcel courier DHL, thereby leading to fragmentation due to different private actors who focused on various aspects of stockpiling such as procurement, storage, and information technology. All these actors were also largely focused on prioritising efficiency savings, which failed to account for caring concerns (Hall et al. 2020).

In Canada, the Public Health Agency of Canada, which had been established in the wake of the severe acute respiratory syndrome (SARS) pandemic in 2003 through the Emergency Management Act, continued to oversee the National Emergency Stockpile System (NESS). However, in 2019, the federal government closed three out of the nine warehouses and discarded large amounts of PPE without replacing it or making provisions to that effect. Instead, stockpiling was devolved to the provincial level (Laing and Westervelt 2020), with provinces maintaining the remaining unreplenished warehouses (Silverman et al. 2020). Similarly, the US had not entirely replenished supplies after the H1NI crisis and relied on a network of supply chains which varied very much from state to state (Bhaskar et al. 2020; Handfield et al. 2020; Queen Haywood 2020).

At the beginning of the crisis, it became evident that due to the lack of centralised mechanisms by governments in all three countries to monitor and refresh the stockpiles, the PPE that was in storage was totally unusable. In the UK, for instance, 200 million pieces of PPE turned out to be expired, with over half of all surgical face masks in the national inventory unusable (Channel 4 News 2020), as was also the case in Canada (Laing and Westervelt 2020). In the US, the Centers for Disease Control and Prevention (CDC) recommended using expired N95 masks due to severe shortages (Cohen and Van der Muelen Rodgers 2020). These problems were linked in part to the fact that the logistical logic and just-in-time model applied to stockpiling and preparedness suggested that countries could continuously stock up on PPE during the crisis. For instance, in Canada, most provinces did not plan for a national emergency and therefore assumed that they could offset their lack of PPE on supplies from other provinces (Silverman et al. 2020). The NHS in the UK assumed that it could purchase the supplies that it needed quickly from suppliers abroad. In the US, the entire country had only 1% of the N95 masks that it needed (Bhaskar et al. 2020).

In the immediate aftermath of the crisis, all three countries allowed some use of expired PPE, but they were particularly reliant on third countries to provide a fresh supply (Channel 4 News 2020; Cohen and Van der Muelen Rodgers 2020; Laing and Westervelt 2020). However, because of worldwide shortages of PPE, countries soon introduced counter-protectionist measures which made shortages more acute. This led to the establishment of home-grown industries and repurposing of private industries to make PPE (Gereffi 2020).

It is therefore evident that preparedness failed because of the failure of national strategic stockpiles, which in all three countries had been decentralised, chronically underfunded, and diminished in part because of reliance on outsourcing in all three jurisdictions which prioritised cost-effectiveness of storage and relied on logistical logics of just-in-time delivery and the assumption that countries would be able to continuously stock up on PPE. Additionally, the geographical reorientation of (medical supply) value through the WTO regulations that encouraged geographical reorganisation of supply chains into high-value and low-value regions (Gereffi 2020; Meyer 2020) contributed to the problem.

## Reproducing Neoliberal Logics: Securing Supply Chains through Emergency Procurement

As supply chains failed, the shortage of PPE disproportionately impacted on perceived ‘low-value’ intimate care settings within national contexts, such as care homes. This was in part due to the lack of specific attention for care workers outside of the hospital and acute health care settings in the response to the crisis (CIHI 2020). In trying to examine how this played out, we sketch out the different country responses adopted to address the resulting shortages. In each case, responses involved mobilisation of significant financial resources to fund public procurement and, in parallel, an effort to reorient supply chains to local sites of production by incentivising domestic manufacturing. In this section, we focus primarily on the former, and return to nationalisation efforts in the ‘Two Different Regimes, Same logic Section’.

## Shoring Up Supply through Emergency Procurement

Given the geographies of value that characterise the production of different types of medical supplies, all three countries engaged in efforts aimed at shoring up global supply chains for procurement of PPE and other goods. Of the three, Canada is the most heavily reliant on imports of medical supplies. However, even the UK and US rely on global supply chains—for nearly all PPE in the case of UK, and a third of the total US medical supply needs, especially PPE (Leibovici et al. 2020). Beyond PPE, all three countries rely on global markets for components and raw materials needed for domestically manufactured medical products, making foreign procurement integral to government responses despite calls for renationalisation of supply chains.

In all three countries, these responses were facilitated through emergency legislative measures that faced very little scrutiny from the public. In Canada, for instance, the federal government adopted a series of emergency funding packages and passed legislation mandating unlimited spending to acquire medical supplies, including PPE, and assuming wide powers to address supply chain obstacles^5^. A temporary procurement policy was adopted which suspended the requirements for competitive bids or tendering, and increased spending limits (Treasury Board of Canada Secretariat 2020a). The Canadian government mandated expedited sales authorisation of medical devices for use in relation to COVID-19, thereby relaxing rules around approved vendors (Treasury Board of Canada Secretariat 2020b). In the UK, the Coronavirus Act 2020 made similar provisions, and procurement rules were also loosened. In the US, the Trump administration invoked the Defense Production Act 1950 (DPA), mobilising resources to address supply chain disruptions, albeit sporadically and narrowly (Congressional Research Service 2020). The US Congress also adopted emergency legislation to address procurement needs.^6^

While the specifics of global procurement drives varied across the three cases, some significant parallels emerge in relation to the processes that were foregrounded, actors who were prioritised, and problems that ensued.

In all three countries, governments turned to private firms in order to source PPE directly from China in ways that encouraged privateering through lowering of minimum standards for goods that would then be rendered unusable, corruption through preferential treatment for middlemen, and a reliance on patronage. In Canada, private consulting firms (e.g. Deloitte) and multinationals (Bollore Logistics, Amazon, etc.) were quickly hired to help identify suppliers and to facilitate logistics (Public Services and Procurement Canada 2020). In the UK, botched or private deals leading to enrichment by insiders were widely reported; in one such deal a business consultant acting as a ‘middleman’ between an NHS supplier and PPE manufacturers made £21 million on a deal to supply PPE (Adkins 2020). As in Canada, delays and product deficiencies were also a problem in the UK (Mason et al. 2020). In the US, the foreign procurement efforts were coordinated by the Supply Chain Stabilization Task Force created by the Federal Emergency Management Agency (FEMA) and the US Department of Health and Human Services. The task force’s ‘Project Airbridge’ was led by Jared Kushner, the son-in-law of then-US President Donald Trump, together with private logistics and large distribution firms. This led to procurement of products that failed safety standards, resulting in huge waste of government resources (Adkins 2020; Antle 2020; Mason et al. 2020; National Audit Office 2020).

The major capital investments that governments were prepared to make to stabilise supply chains and develop local capacities to respond to pandemic and other crisis events in the future suggests a partial shift away from the neoliberal orthodoxy. At the same time, in all three cases the crisis management response in the pandemic’s chaotic first wave tended to reproduce at least some well-rehearsed neoliberal blueprints by prioritising public–private partnerships with large logistics and distribution firms or engaging in wholesale outsourcing of government functions to private consultancies. Far from creating efficiencies, this approach was plagued by lack of transparency, insider deals, and enrichment by large corporate actors and government officials, often resulting in delays and defective products.

## Uneven Geographies of Care-less Supply

Moreover, even when this style of crisis management succeeded in procuring supplies, these reached different care settings and different care workers in an uneven way, with significant consequences for delivery of day-to-day care, safety of workers, and morbidity rates among residents and staff (Chidambaram et al. 2020; CIHI 2020; Comas-Gerrera et al. 2020). In all three cases, the policy responses narrowly conceptualised care delivery and settings, with frontline work in a pandemic being primarily that of acute health care carried out in hospitals (Daly 2020; Jackman et al. 2020). For instance, Poon et al. (2020) identified that in the UK between January and April 2020, there were 24 PPE-related guidance notes issued, with only one of them (27 April) specifically on health and social settings. Before that, the managers of health care workers had no specific guidance for care homes to use masks (Rajan et al. 2020). Institutional settings like care homes and nursing homes where day-to-day intimate caregiving takes place were not given much attention in national preparedness strategies, or during the first crucial phase of the pandemic response, with care workers being perceived as a secondary front line (Nyashanu et al. 2020; Hoernke et al. 2021).

Thus, while PPE shortage was a problem across all health care settings, it became critical in long-term care facilities, especially those privately operated, where it was most heavily rationed (not least because of the reliance on lean management techniques) and systematically denied to frontline workers. In Canada, for example, reports by the military (which was called to assist in the provinces of Ontario and Quebec) and lawsuits filed by unions (Jackman et al. 2020) have revealed PPE being kept under lock and key, workers being required to go through complex and lengthy procedures to access it, and access being unevenly distributed, with the most vulnerable workers, those working in closest proximity to infection, not being prioritised over doctors and supervisors, etc. (Lippel 2020). Similarly, in the US, despite the federal government’s promise to supply all US nursing homes with two weeks’ worth of PPE in early April 2020, many nursing homes reported that they never actually received adequate PPE through this initiative. Instead, shipments either failed to arrive or contained inadequate or low-quality supplies (Rau 2020). Surveys carried out in July and August 2020 (McGarry et al. 2020) indicated that one in five long-term care/nursing home facilities still experienced severe shortage of PPE during this period, with the most acute shortages in for-profit facilities, suggesting that cost saving played a role in decisions about investment in PPE. The situation was very similar in the UK, where research suggests that the government almost entirely prioritised the NHS at the expense of the social care sector (Daly 2020).

The opening of public coffers to address the collapse of medical supply chains and the problem of PPE shortage created an opportunity to transform how procurement of these supplies is handled, and how they are distributed to facilitate care relations during a crisis but also in everyday practices of care. On the one hand, the states’ willingness to commit significant financial resources to secure supplies does signal a shift away from more traditional neoliberal scripts. However, as we have shown here, problems of transparency, corporate and political enrichment, and inefficiency plagued the responses adopted in all three countries. This was the case in relation to global and local procurement, and in the drive to bolster national production, all of which, especially in the first months, saw significant transfer of public funds to private, often multinational firms.

Crucially, while the safety of frontline workers was given so much prominence in public discourse and in how states’ responses were framed and justified, the rates of infection and death among nursing staff and home care workers reported in the three countries suggest that care and care workers, especially in the secondary frontline of long-term care, were given low priority. Thus, to some extent, these moves reproduced the care-less neoliberal logics that gave rise to the crisis in the first place, in a manner consistent with what Naomi Klein terms ‘disaster capitalism’ (Klein 2007), or what others have described as a technique of governance which reinscribes and reproduces hegemonic orders and circumscribes the ways in which the latter can be challenged and what opportunities for transformative change are actually possible in light of crisis (Branick 2020, with reference to Otto 2011; Griffin 2015).

## Challenging Neoliberal Logics? Some Proposals for Supply Chain Resilience

Given the failures described above, there have been numerous suggestions on how best to approach pandemic preparedness, with key emphasis being placed on improving the resilience of medical supply chains. In this section we review these suggestions and reflect on their implications for the geo-legality of preparedness, and the extent to which they indeed challenge the neoliberal logics that contributed to the failure of supply chains during the pandemic. We start with calls for developing local supply chains or reshoring of goods that national companies produce abroad to reduce reliance on imports, which, as we have seen, have been partly actioned through integration into national pandemic responses. We also look at proposals around increased or more efficient stockpiling of medical supplies, especially through better use of technological solutions such as blockchain and artificial intelligence (AI) and those focused on regulatory changes through greater coherence in medical supply standard setting and clarification of WTO rules. Finally, we also draw attention to other proposals, such as those focusing on supply chain sustainability through ‘commons’.

## Reshoring and Developing Local Supply

Calls for relocating manufacturing activities back to the home country (i.e. ‘reshoring’) or sourcing of supplies from countries that are more proximate, preferably those with closer geopolitical ties (i.e. ‘near-shoring’), have been part of broader and longer-term nationalistic agendas that seek to limit countries’ dependence on China (Cutler 2020; Gurvich and Hussain 2020) and minimise vulnerability to import bans or supply chain issues in the midst of a crisis.

Parallel to global sourcing efforts that we described in the previous section, all three countries in our analysis invoked the need to bolster or develop national medical supply chains. At the start of the pandemic, these efforts were largely linked to national security concerns through initiatives such as ‘Project Defend’ in the UK which identified medical supply chains as being vulnerable to foreign interference from third states such as China (Reuters 2020). In the US, the nationalisation agenda was framed through the lens of security by both the Trump and Biden administrations. The National Strategy for the COVID-19 Response and Pandemic Preparedness adopted on Biden’s second day in office in January 2021 promised to pandemic-proof the country through a “resilient, domestic public health industrial base…a flexible supply chain…and [expanded] American manufacturing capability where the United States is not dependent on other countries in a crisis” (Biden 2021, 73). To put this into effect, the administration’s executive orders set out to ensure robust stockpiles and sustainability in supply chains though a “buy American” policy whereby government agencies are to “procure goods, products, materials, and services from sources that will help American businesses compete in strategic industries to help America’s workers thrive” (The White House 2021a, 4475; see also 2021b). In Canada, the ‘Made in Canada’ strategy introduced in March 2020, has been bolstered by several long-term procurement contracts that allowed local firms to make significant capital investments (beyond short-term retooling) such as building factories and developing specialised equipment (Harris et al. 2020; ISED Canada 2020). A number of testing and certification facilities have been built across the country^7^ to address the logistical problems that manufacturers who retooled to produce PPE faced in the first few months of the pandemic (Canadian Press 2020).

Despite these moves, questions about costs and efficiency have already resurfaced. In more recent US communications and strategic statements, for example, focus on PPE—so prominent at the start—seems to have given way to focus—insofar as medical goods—on essential medicines, pharmaceutical components, and high-value medical supplies.^8^ Similarly, in Canada, concerns have been raised about the long-term feasibility of homegrown production of PPE given its higher costs and, at the later stages in the pandemic, lower demand (Harris et al. 2020).

## Better Stockpiling and Technology-Enhanced Sourcing

Given that questions about the feasibility and desirability of nationalising and reshoring supply chains remain, other proposals have focused on improving the current system of preparedness through increased and smarter stockpiling of medical supplies, as well as digitised, transparent, and more responsive supply chain management.

With respect to stockpiles, new models have been proposed wherein buying is centralised and national stockpiles act as a conduit through which regional supply chains are ordered to ensure that goods do not expire while in storage (Bhaskar et al. 2020; Handfield et al. 2020). Others have also called for regional stockpiling which would lead to regional hubs of medical supplies that could be rapidly deployed in a crisis. Previously, the Association of Southeast Asian Nations (ASEAN) countries with Japan had made a similar effort for avian influenza (WHO 2007). In the current COVID-19 crisis, we saw regional funds that were used to procure medical supplies jointly in the European Union and the South Asian Association for Regional Cooperation, which set up an emergency COVID-19 fund for member countries (Bhaskar et al. 2020). Regional stockpiles are part of a broader global health security approach, although they recognise that previous efforts have not been successful due to geopolitics (Katz and Standley 2019).

Improving supply chain efficiency through digital technological solutions such as blockchain and AI has also received much attention. These technologies follow on from previous solutions such as barcodes used in mass shipping containers, which galvanised supply chains in the twenty-first century. For example, AI systems could be used to map inventory and production in real time, streamlining processes through greater automation and resulting in greater transparency (Bagayoko et al. 2020; Bhaskar et al. 2020). It is said that, in a crisis, a blockchain would allow all parties greater visibility of the entire supply chain amidst fluctuating demand and supply, where different countries may experience crises differently. This visibility would allow parties to obtain information in real time, which would lead to fairer negotiating, greater transparency due to more accurate information, and, finally, reduced delays between the ordering process and delivery of products (Bhaskar et al. 2020). Huge epidemiological data sets could determine which regions or countries needed medical supplies, streamlining orders (Bhaskar et al. 2020).

## Greater Regulatory Coherence

During the pandemic, countries struggled to scale up their supply of medical goods due to different regulatory standards that stood in the way of efforts to divert supply chains. Given this, there have been increased calls for greater regulatory coherence amongst countries in order to ensure that medical supplies can be repurposed between jurisdictions that may have different standards (Gereffi 2020). Others have also called for the relaxation of strict product liability during crises which leads to different standard setting in countries (Product Law Bulletin 2020). Internationally, some scholars have called for changes to the WTO deeming it to be a particularly good avenue to creating common core standards for regulation of medical supplies (Hoekman and Sabel 2019). Other proposals for WTO reform that may impact on medical supply chains include making the notification of emergency authorisations simpler, creating a committee to handle crises and allowing the use of open-source patents during a crisis so that other manufacturers can step in (Meyer 2020; Wolfe 2020).

More recent proposals are pushing for supply chains to be considered as an essential element of preparedness within proposals for a new Pandemic Preparedness Treaty (WHO 2021). Specifically, proposals call for agreeing to pre-specifications on quality assurance that would lower standards in the event of a crisis, thereby allowing smaller players to enter the market more easily, greater strategic investment in infrastructure, increased financing for preparedness and open data systems, and using regional blocs to harmonise purchases in order to ensure that the just-in-time model can work better during a crisis (Secretariat of the IPPR 2022).

## Challenging Neoliberal Geo-legality of Preparedness and Supply Chains?

Like direct government intervention and increased spending that have been key features of crisis management, the moves towards reorienting supply chains and geographies of production appear to challenge the trade-oriented neoliberal status quo to which global supply chains have been so central. Indeed, nationalisation of supply chains has been advocated by scholars critical of the localised social and environmental impacts of global production (Cutler 2020). However, in our three country studies, the benefits of these reorientation efforts have so far not been widely distributed among different local economic actors/producers, with larger firms being better positioned to take advantage of the public financial supports. Moreover, the practical realities of reshoring and nationalising supply chains are quite complicated and questions about costs and efficiency have already resurfaced in the US and Canada alike. Given that components and raw materials that go into both the basic and the more specialist medical products are also globally sourced, moves towards reshored production are unlikely to fully disrupt global circulation of raw materials (Hopewell and Tafel 2020).

While smarter stockpiling and technology-enhanced sourcing solutions promise to improve transparency and communication in the just-in-time model, they work on the assumption that the problems of supply chains were largely created by intermediaries (middlemen, procurement consultants, etc.) and so the problem will be resolved when technology connects end users such as hospitals and manufacturers and is comprehensive enough to survey the entire eco-system of supply chains using more reliable predictive modelling chains. However, while the use of technology can help with purchasing efficiencies, inventory oversight, and better distribution and thus help avoid depletion and waste (i.e. supply expiry), it does not address the problem of sufficient public funding which was one of the key factors that contributed to the stockpile failures during this latest pandemic. Nor does making stockpiles in northern countries more robust necessarily guarantee pandemic preparedness at a global scale, given limited resources and capacities in lower income countries.

The calls for regulatory coherence also miss the broader systemic problems inherent in governing supply chains through a neoliberal framework, prioritising technical fixes that strengthen aspects of law without addressing the wider logic that seeks to ensure minimal disruption to trade. Many of these models appear to aim at making existing processes more efficient without critically reflecting on and addressing the unique nature of medical supply chains and the centrality of their care-facilitating function.

The insufficiency of the above proposals, combined with questions of equity in access and capacity, have prompted calls on states to think beyond this particular crisis and to instead hone in on the systemic problems that underlie medical supply issues. As they point out, the problems of unfair competition at the expense of cheaper generics and the proliferation of complex networks that rely on middlemen have been endemic to the neoliberal model of global medical supply. Therefore, possible reforms need to focus on developing medical products capable of being reused that include free and open hardware which enables other manufacturers to make them easily in moments of crisis and challenges broader ideas around intellectual property ownership by companies who currently make medical supplies (Miller et al. 2021). This could be through the use of patent pools in which patents are shared for a common objective or creative common licenses which enable a larger number of manufacturers to make medical supplies. Once goods are produced, these should also be distributed equally through pool mechanisms as opposed to competitive procurement in order to ensure that countries get them based on need as opposed to resources during health emergencies (Sinha et al. 2020).

These commons-based proposals draw attention to that fact that future approaches to preparedness and medical supply chain organisation and governance must address the unequal geographies and hierarchies of health care which currently exist in local and global domains. Indeed, we argue that because these geographies and hierarchies of care are not acknowledged by reshoring, technology-facilitated stockpiling, and greater regulatory coherence proposals, these proposals only partly challenge how the approach to supply of medical goods has been handled so far; namely, as goods devoid of the care they facilitate.

Therefore, these proposals continue to ignore the role of supply chains as active in constructing and delimiting the conditions in which provision of care is made (im)possible. While the support of populations and life is an inherent part of logistical logics (albeit in accordance with the neoliberal logics of geoeconomics and geopolitics, and alongside reproduction of those systems), as our discussion highlights, pandemic preparedness and responses structured through these logics have managed to sideline some care settings and care providers in a manner that reflected and reproduced systemic (gendered, racialised, intersecting) inequities and hierarchies of (care) value. In light of this we ask: should not supply chains that are integral to provision of care be governed in a manner that puts care (rather than efficiency and short-termism) at the centre? Below, we consider what centring care reveals about the current proposals, and how a feminist ethic of care might serve as the basis for providing alternative organisational logics in the supply chain of medical products.

## Bringing Care in: The Ethics of Care Perspective

The feminist ethics of care perceives care as a moral orientation and a set of practices that “includes everything we do to maintain, continue and repair our world so we can live in it as well as possible” (Fisher and Tronto 1990, 40). At its core is a relational ontology of interdependence and the premise that the ability to give *and* receive adequate care is central to human well-being (Robinson 2013), and indeed, constitutive of life itself (Hoppania and Vaittinen 2015). Crucially, care is not something we can do alone—it is a collective process that requires reciprocity and solidarity (Federici 2010). As such, according to care ethicists, how we give and receive care, and how we care *with* each other are both moral and political questions that should inform our interactions at every level (Tronto 2013). The extent to which care giving and receiving is facilitated or inhibited by practices, institutions, structures, and discourses operating at multiple sites and scales is a question of justice/injustice (Robinson 2013).

Feminist scholars have applied ethics of care to critically scrutinise the injustices that ensue when care (relations, practices, and institutions) is reorganised in accordance with neoliberal policies (and logics) and under conditions of globalisation. Similarly, care ethics also provide a critical perspective on policy and institutional responses that may not be directly related to care, but which may be otherwise ‘care-less’ or have collateral implications for care relations across a range of sites. As we have argued above, the neoliberal logics according to which preparedness has been structured, and medical goods circulated, up to now is fraught with problems that need a more robust solution than a return to the status quo, which is an objective associated with mainstream (“rationalist”, “utilitarian”, and “militaristic”) crisis management approaches that fail to produce meaningful change (Branicki 2020, 872). To what extent are the recent proposals focusing on bolstering supply chain resilience facilitating care giving and receiving or, at the very least, challenging the ‘care-less’ logics on which the approach to pandemic preparedness has been based thus far?

## Evaluating Proposals Through the Ethics of Care Perspective

The proposals that emphasise nationalisation of medical supply chains through reshoring and bolstering of domestic production can be seen as facilitating care and caring because such approaches can lead to more efficient and secure supply chains, which are physically more proximate to the care sites at which they are required. Such approaches can also facilitate caring in a sense that they sometimes emphasise responsible business practices and are said to respond to problems of labour exploitation and environmental sustainability by moving production to jurisdictions where oversight is easier. What nationalisation or reshoring proposals tend to miss, however, is the complexity of care in the context of global supply chains and the relations (of care) with those people (largely women) who are making medical supply goods, be it abroad or locally. While they promise a more secure supply, their sustainability—something that is highlighted—is questionable, given the heavy reliance on global circulation of raw materials needed to produce even those supplies that are or can be manufactured locally in countries we examined here (with implications for jobs abroad), or the focus on automation in production in order to make local production profitable for firms, with its necessary implications for jobs. As we have seen, reshoring practices during the pandemic saw the transfer of public funds to benefit certain actors (i.e. large corporations), and ultimately proposals that emphasise reshoring as a future approach, especially those articulated through nationalist discourse, sidestep the question of how we could care better, and more equitably. Organising medical supply in a way consistent with the ethics of care would require that we think about the kinds of products, who is making them, and who is going to use them in which setting and for whose benefit. This would require broader geographical solidarities that go beyond narrow national interest and focus on the intimate needs that caring demands, especially during global public health crises.

Current attempts to improve stockpiling through technological solutions operate to preserve the status quo by attempting to build the same system back, but more efficiently; as such, they do not fully challenge the neoliberal logics that undermined stockpiling up to now. This can be seen for instance with the preoccupation with public–private partnerships in order to make stockpiles more viable (and cost-effective?) in the long term (Katz and Standley 2019). Given that costs and viability continue to be major considerations, there is a danger that, over time, it will no longer be economically feasible to stockpile at all, for instance. Also, as with the issue of efficiencies related to local supply, the discussions around stockpiles and technology tend to focus on *things* as opposed to people at the core of the care systems, those who care and receive care.

As for as regulatory coherence, states are divided about the way forward. Take our three case study countries. The US and UK are seeking local solutions to PPE supply issues and take less interest in coordinating global legal efforts, while Canada is leading globalised efforts within the WTO to reassert the WTO’s role (if in a modified fashion). Thus, the logic that informs these various positions is very much driven by states’ (political-economic, strategic, security, etc.) interests and not the interests of care. If care were the guiding principle, the current neoliberal model in which we have different regulatory standards would be replaced with a move towards harmonisation of standards for caring products such as PPE as opposed to differentiated standards that are relaxed during a crisis so that the cost of retooling is not exorbitant when it does become necessary. That kind of move would be more consistent with a focus on carers and care receivers *everywhere* as being of equal value.

A feminist ethic of care demands that all attempts at reforming the system foreground the intimate practices and relations needed to care effectively during public health crises. Current attempts to resolve supply chain issues will fail because they fail to focus on the materiality of care provision that is necessary in order to prepare and respond to public health crises. Therefore, many of the solutions fall short: reshoring proposals end up focusing on the essential *products* such as PPE that enable caring, the regulatory controls sidestep caring and instead offer technical fixes on how the current system could support a just-in-time model without considering the wider inequity in terms of how that system may be deficient for those at the bottom of the caring chain, and technological solutions tend to decentre humans from the process of caring. A feminist ethic of care would also entail less logistically driven top-down solutions and more radical demands for systemic changes that place carers at the centre of any proposals.

## Concluding Remarks: Feminist Care Ethics for Medical Supply Chains

To ensure that future pandemics do not disproportionately impact those who are most vulnerable (be they care providers or recipients, or those who are engaged in production of supplies), medical supply chains have to be reconsidered and governed according to logics other than those of logistics and efficiency. We suggest that feminist approaches provide some such alternative logics, with those that emphasise care being particularly promising.

A feminist strategy of preparedness necessitates that we centre caring relations and care ethics over the logics that emphasise extraction of profit and efficiency insofar as production, distribution, and allocation of medical supply. Doing so means also being cognisant of the plethora of ways in which the local (and intimate) and the global are currently entwined and crafting solutions that are most likely to operationalise a system of global medical supply that is equitable, based on values of common good, solidarity, and sustainability.

As we have noted above, developing local (national) manufacturing, storage, and distributional capacities to respond to crises might be one step to ensuring that care can be delivered in conditions of crisis/emergency without the need to compromise the safety of those on the frontline or prioritise some care settings at the expense of others. At the same time, local solutions must also be attentive to their potential *global distributional and care impacts*. Thus, a move to national manufacturing in countries that have previously outsourced low-value medical goods through global supply chains can partly address their current overreliance on global suppliers, but the same move can also destabilise sectors and economies that have specialised in the production of low-value PPE, leaving workers in the global value chains to ultimately bear the burden of these shifts. Thus, we argue that attempts to reshore the making of PPE must centre reparative justice which attempts to mitigate against countries who have traditionally borne the risk of off-shoring from the changes to patterns in production. This may for instance involve clarifying the remit of international assistance under any reforms in global health to include not only preparedness as a positive action but also the impact of shifts in preparedness strategies.

Moreover, nationalisation does not in itself ensure that caring relations are centred in local manufacturing, not least given that erosion of work conditions, practices of workplace fissuring, and informalisation are widely documented global tendencies. So long as efficiencies and profits remain central to the manufacturing of medical supplies, a shift to national production would not necessarily resolve labour-related care deficits in local supply chains or in how the supply is distributed across care settings in an emergency. As such, diversification of supply chains through a blend of local and global sourcing might be a strategy that more effectively balances the risks (and consequences) associated with overreliance on global supply on the one hand and wholesale nationalisation on the other hand. But even then, a care-centric approach must focus on the conditions in which the supply is produced (anywhere) and how it is circulated across the care ecosystem both in normal times and during an emergency. The former question—of labour conditions—is rarely addressed in any of the proposals for medical supply chain resilience, but it is a key question if care were the ethic according to which we reimagine pandemic preparedness and the role of medical supply chains within it.

Thinking about preparedness with care for local–global relations not only means addressing the problem of adequate supply and ability to *equitably* respond to health emergencies such as global pandemics across the national care ecosystems; it also means preparedness and distributional equity at the global scale. Namely, a care-centric approach to preparedness means facilitating the ability to respond for countries that currently lack capacities to rapidly shift to national production or engage in aggressive procurement. This is especially important if we are to centre diversification of supply chains as a response that best balances global and local concerns. In this context, *sharing* (resources, knowledge, technology) emerges as a key element of a care-centric approach. Through the prism of care, the know-how and technology required to produce basic (generic) medical supplies are common-pool resources of the global human community that ought to be available to institutions or manufacturers in any country.

Additionally, questions of sustainability such as the distances that goods move, the overreliance on single-use products for PPE, the energy spent creating either goods or even technological fixes such as blockchain all need to be critical to the ways in which we think about medical supply chains in the future.

## Footnotes

1 There is a vast feminist literature on global supply chains and global value chains, which we do not directly engage with here, but which provides an important backdrop to our understanding of global supply and value chain dynamics, particularly their role in (re)producing inequalities at the global scale. Here, we are using the term ‘global supply chains', as we are interested in their role in the supply of and circulation of medical goods as part of global and national pandemic preparedness strategies.

2 Scholars have pointed out that supply chains—or global value chains—are not simply economic structures but are legally constituted and, in turn, have implications for legal governance. See, for example, (IGLP 2016).

3 Ann Stewart (2011), for instance, has examined supply chains that circulate food and other necessities of life through the prism of care relations, proposing that foregrounding care (ethics) in global economic relations is necessary to global (gender, in its various intersections) justice. Our argument in this paper (especially in Sect. 5) develops partly along these lines, albeit applying ethics of care to global *medical* supply.

4 In looking at the literature on preparedness, we make two observations. Firstly, responses to failures of preparedness were geared exclusively at certain kinds of goods and services. For instance, countries rapidly constructed additional hospitals to address capacity issues (Yanakieva 2021; i.e. the UK’s Nightingale Hospitals) and easily scaled up the production of ventilators. By contrast, the most prevalent problems that the literature on preparedness describes were centred on PPE. Secondly, the gendered nature of the lack of preparedness is evident when we compare it with precautionary, and disproportionate, military investments. Pandemic preparedness is costly, because countries must constantly maintain surplus stocks (Braga 2021; Timmis and Brüssow 2020). Invariably, the neoliberal logic of just-in-time supply chains has affected how states view this kind of preparedness, with legal frameworks and responses being particularly illustrative.

5 Bill C-13: *An Act respecting certain measures in response to COVID-19*S.C. 2020, c. 5.

6 The Medical Supply Transparency and Delivery Act, H.R. 6711/S. 3627 (2020) was adopted on 29 April 2020 and was inserted in the Health and Economic Recovery Omnibus Emergency Solutions (the HEROES) Act, H.R. 6800 (2020) on May 12, 2020.

7 For example, the PPE Testing and Accreditation Lab, which was opened in Vancouver, BC, in Fall 2021.

8 For example, the Building Resilient Supply Chains, Revitalizing American Manufacturing, and Fostering Broad-based Growth supply chain review report summarizing the first hundred days of the Biden administration’s response makes only a single reference to PPE (The White House 2021c).
